# The impact of COVID-19 on surgical training: a systematic review

**DOI:** 10.1007/s10151-020-02404-5

**Published:** 2021-01-28

**Authors:** C. Hope, J.-J. Reilly, G. Griffiths, J. Lund, D. Humes

**Affiliations:** 1 Division of Medical Sciences and Graduate Entry Medicine, University of Nottingham, Derby, DE22 3NE UK; 2 Queen’s Medical Centre, Nottingham University Hospitals NHS Trust, Nottingham, NG7 2UH UK; 3 Ninewells Hospital, Dundee, UK; 4 Division of Epidemiology and Public Health, School of Medicine, University of Nottingham, Nottingham, UK; 5 Nottingham Digestive Diseases Centre, School of Medicine, University of Nottingham, Nottingham, UK; 6 National Institute for Health Research Nottingham Digestive Diseases Biomedical Research Unit, Nottingham University Hospitals NHS Trust, E Floor West Block, QMC Campus, Nottingham, NG7 2UH UK

**Keywords:** Surgery, Medical education, COVID-19, Coronavirus 2019, Surgical training

## Abstract

**Background:**

Coronavirus disease (COVID-19) has caused global disruption to health care. Non-urgent elective surgical cases have been cancelled, outpatient clinics have reduced and there has been a reduction in the number of patients presenting as an emergency. These factors will drastically affect the training opportunities of surgical trainees. The aim of this systematic review is to describe the impact of COVID-19 on surgical training globally.

**Methods:**

The review was performed in line with Preferred Reporting Items for Systematic Reviews and Meta-Analyses (PRISMA) guidelines and registered with the Open Science Framework (OSF). Medline, EMBASE, PubMed and the Cochrane Central Register of Controlled Trials were searched.

**Results:**

The searches identified 499 articles, 29 of which were included in the review. This contained data from more than 20 countries with 5260 trainees and 339 programme directors. Redeployment to non-surgical roles varied across studies from 6% to 35.1%. According to all of the studies, operative experience has been reduced. Knowledge learning had been switched to online platforms across 17 of the studies and 7 reported trainees had increased time to devote to educational/academic activities. All of the studies reporting on mental health report negative associations with increased stress, ranging from 54.9% to 91.6% of trainees.

**Conclusions:**

The impact of COVID-19 on surgical trainees has been experienced globally and across all specialities. Negative effects are not limited to operative and clinical experience, but also the mental health and wellbeing of trainees. Delivery of surgical training will need to move away from traditional models of learning to ensure trainees are competent and well supported.

**Supplementary Information:**

The online version contains supplementary material available at 10.1007/s10151-020-02404-5.

## Introduction

The coronavirus disease (COVID-19) continues to have a worldwide impact. To date, there have been over 16 million cases and over 640,000 deaths reported [1], with some countries still reporting increasing numbers of cases. This global health crisis has placed huge demand on healthcare systems and the prioritisation of surgical services has shifted. Non-urgent elective surgical cases have been cancelled [2, 3], outpatient clinics have dramatically reduced [4] and there has been a reduction in the number of patients presenting as an emergency [5]. Examinations, courses and conferences have been postponed on an international basis across specialities [6, 7]. The reprioritisation of health services, redeployment of staff to COVID-19 wards and decrease in operative volumes may have a significant impact on surgical training and the mental wellbeing of surgical trainees.

Whilst all specialty training has been affected it is perhaps craft specialties which have been most affected, with the lack of procedural training opportunities. Only the most urgent elective cases have been performed and conservative management is increasingly being recommended for some emergency presentations such as appendicitis [8]. Furthermore, to minimise operative time and the risk of COVID-19 transmission intraoperatively, senior surgeons are now performing more of the emergency cases and training opportunities are further reduced [9].

Moving forward, there is a need to quantify the scale of disruption to surgical training to mitigate the adverse effects of lost training opportunities and deficiencies in experience. The aim of this systematic review is to describe the impact of COVID-19 on surgical training globally.

## Materials and methods

### Protocol registration

This systematic review was performed in line with the Preferred Reporting Items for Systematic Reviews and Meta-Analyses (PRISMA) [10]. The protocol is available on the Open Science Framework (OFS) at https://osf.io/xz5h8/?view_only=d2f52ec92c464029ad0cdea5028f547c.

### Eligibility criteria

The review sought to identify papers evaluating the impact of COVID-19 on surgical training worldwide. All surgical specialities were included. All types of published research articles were included with no restrictions on the language of or date of publication.

### Exclusion criteria

1. Letters, correspondence and editorial reviews were not included.
2. Obstetrics and gynaecology was not considered a surgical specialty for the purpose of this review.

### Information sources, search and study selection

MEDLINE Ovid, Embase Ovid, PubMed and the Cochrane Central Register of Controlled Trials (CENTRAL) were searched electronically from January 2020 up to 31st August 2020 using a mixture of keywords and MeSH terms (Supplemental Fig. 1). The reference lists of included studies were searched for further eligible studies.

The title and abstract screening was performed by two review authors (CH and JJR) independently and in duplicate. Potentially eligible studies were evaluated in full text to identify studies meeting the inclusion criteria. A third reviewer opinion was sought in the event of disagreement.

### Data collection process

The data were extracted from the studies using a pre-designed proforma, independently and in duplicate (CH and JJR). Study authors were contacted if further information was required for the included studies. The primary outcome measure was the impact on surgical training. Impact could be measured in terms of operative cases, changes to patient contact, redeployment, extension to training or trainee views on changes to training quality. Country of origin, study size, surgical specialty, methodology and outcomes were recorded.

### Risk of bias in individual studies

Methodology checklists for both cohort and case–control studies were reviewed, and used to critically appraise and grade the evidence of included studies. Quality was assessed using the Newcastle–Ottawa scale [11].

### Synthesis of results

The results section was divided into themes arising from the studies: operative impact, non-operative impact, redeployment, educational/academic impact and personal impact. A meta-analysis was not performed due to heterogeneity in study design and differences in training specialty and training system.

## Results

### Study selection

**Fig. 1:**
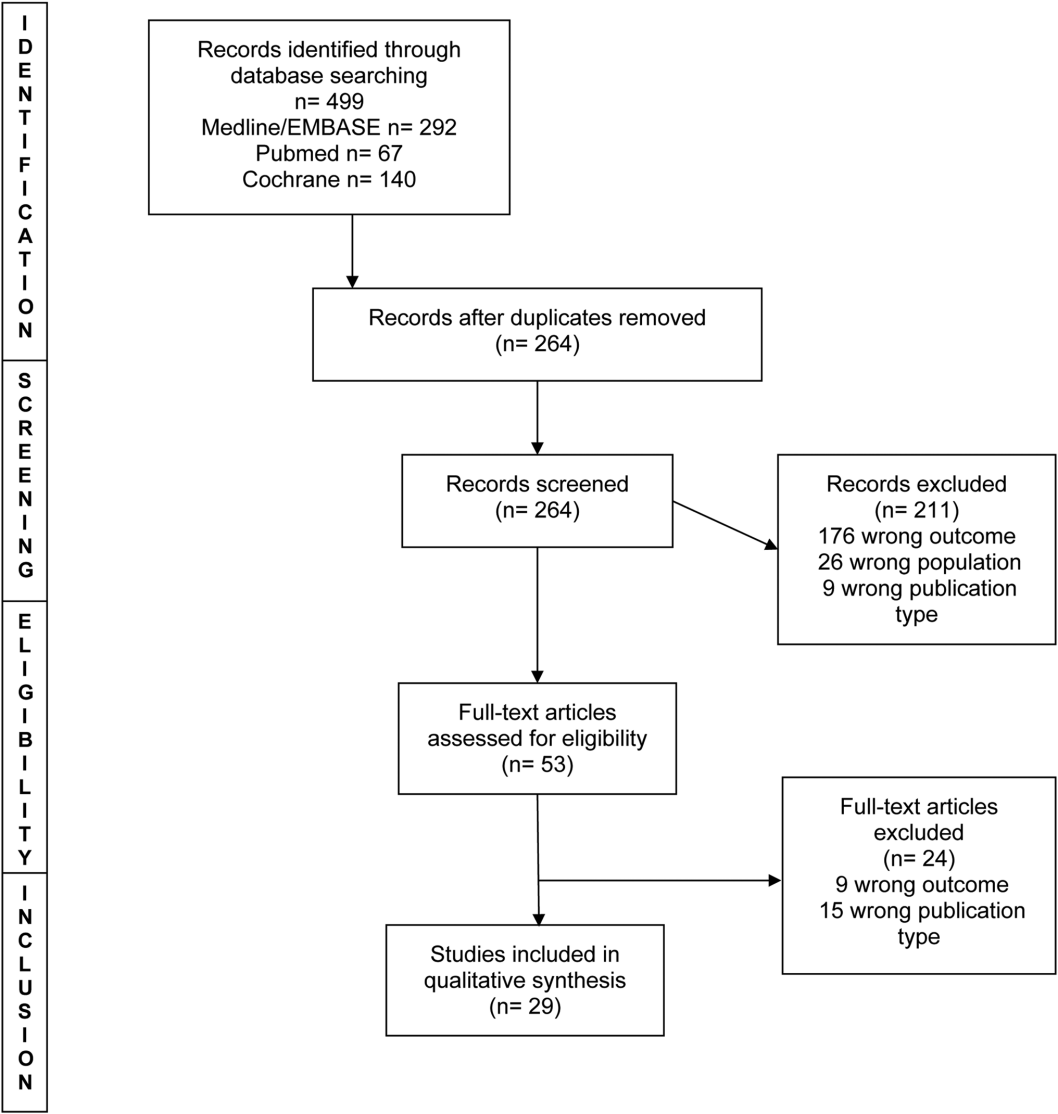
PRISMA flowchart

The searches identified 499 articles (Fig. 1). The main reason for exclusion on title and abstract screening was wrong outcome or wrong population. Twenty- nine studies met the inclusion criteria after full-text review (Table 1). These studies included data from 5260 trainees and 339 programme directors. Thirteen of the studies were from the United States, 6 were from European countries, 1 from South America, 1 from Pakistan and 1 from India, and 7 included data from multiple countries. Twenty- six of the studies were surveys including 5 national surveys across 8 surgical specialties. The survey responses by trainees across different specialities and countries reflect a negative impact of COVID-19 on surgical training. High proportions of trainees felt that the pandemic had adversely affected training [12–14]. We present findings by themes emerging from the included studies.

### Operative impact

All of the included studies reported a decrease in the number of operative cases or operative experience available to trainees. The operative logs of 3 general surgery trainees before and during the COVID-19 pandemic in Italy were compared [15]. There was a significant decrease in the total number of operative cases performed by trainees (*p* = 0.033). It was noted that this particularly affected ‘medium-complexity’ operations which would generally be performed by the trainees as first operator. This decrease was explained by the cessation of benign surgical activity and decreases in the number of emergency admissions. A national survey of 351 Italian urology trainees found substantial reductions in trainee exposure to surgical training opportunities [16]. A reduction in diagnostic procedures was reported by 74.1% of respondents: 62.1% in endoscopic surgery, 57.8% in open surgery and 44.2% in minimally invasive surgery. In the United States, 106 urology trainees included in a cross-sectional survey also reported a decrease in surgical volume dependent upon the local prevalence of COVID-19 [13], with 76% of those working in a high COVID-19 area reporting decreased surgical volume compared to 22% in low COVID-19 area (*p* = 0.01). Aziz et al. reported a significant reduction in self-reported operative case volume amongst 1102 general surgery trainees in the United States [17].

According to 70% (26/37) of head and neck program directors across the United States, there was a 50–90% reduction in elective cases performed by fellows, however 81% reported that fellows continued to participate in oncological cases [18]. Impact was minimised as 82% (25/31) of fellows had already met the operative requirements for certification by the onset of the pandemic. In 2 survey-based studies of oral and maxillofacial surgery (OMFS) trainees in the United States, the pandemic had an impact on the scheduling of elective and non-urgent operations [19, 20]. More than 97% reported that all elective cases had ceased and 83.6% reported that the scheduling of urgent and emergency cases had been affected [20]. While 91.7% of surveyed otolaryngology trainees and faculty reported that all elective cases had been cancelled across 22 countries [21]. Eighty-eight percent stated that their operative experience had been affected with an average perceived decrease in experience of 67% [20]. Three studies investigating the impact of COVID-19 on neurosurgical training found a decrease in the volume of operative cases [22–24]. An Italian study of 192 neurosurgery trainees reported that 78.6% had performed fewer operations than before the pandemic and 16.1% had not performed any operations at all [22]. Similarly, 99.5% of 197 neurosurgical trainees across the United States and Canada reported that the number of operations performed at their institution had decreased [23].

The reduction in surgical cases has caused concerns amongst trainees and program directors; a common theme was the inability to meet case number targets. Fifty- eight percent of orthopaedic trainees across 23 European countries were concerned about meeting annual training requirements [25]. Similarly, 60% of urology program directors in the United States were concerned that trainees will not meet minimum case requirements due to the pandemic [26]. Echoing this, the main concern expressed by 148 urology trainees throughout Latin America and Spain was the impact of the health crisis on their surgical learning curve, with all stating that their operative activity had been at least partially affected [27]. Amongst neurosurgery trainees across the United States and Canada, 8.2% (16/197) were concerned that their overall requisite case numbers would be impacted by the pandemic [23]. Khusid et al. investigated factors associated with concerns over operative autonomy [28]. This was defined as concerns over the ability to operate independently as an attending urologist due to interruptions in training caused by COVID-19. Worries about the ability to reach case number requirements, cancellation of elective cases and higher level of training were risk factors for increased operative autonomy concern after the pandemic [28].

Some studies suggested different experiences for trainees at different stages in training. In a national survey of urology trainees in Italy, final year trainees were significantly more likely to report complete suppression of activities (*p* = 0.003) and reduction in minimally invasive surgery (*p* = 0.002) [16]. Amongst OMFS trainees 66% were not concerned with meeting graduation requirements; however, 60% of senior trainees due to graduate in the next few years expressed concerned regarding this [20]. Senior otolaryngology trainees from the United States reported concerns regarding the negative impact of COVID-19 on future job prospects [29].

### Redeployment, non-operative impact and change to working patterns

Redeployment rates were variable across studies, ranging from 46% [30] to 6% [27]. Redeployment was defined as providing non-surgical care to patients or being transferred to a non-surgical specialty. The highest rate was reported by Kapila et al. with 46% of Belgian plastic surgery trainees providing non-specialty support to patients with COVID-19 [30]. The lowest rate was 6%, reported amongst 148 urology trainees across 18 countries [27]. No studies reported that no trainees had been redeployed.

Some studies reported restructuring of the trainee rotas to incorporate altered working patterns. All 5 of the orthopaedic training programmes in the United States surveyed by An et al. had restructured rotas to include periods of clinical duty followed by remote work and self-isolation [31]. Five studies reported a reduction in trainee presence within the hospital [17, 22, 23, 26, 32, 33]. Ninety-two percent (60/65) of United States urology programs reported a formal reduction of trainee presence, with a significant decrease in patient-contact time from an average of 4.7 days per week to 2.1 (*p* < 0.001) [26]. Pelargos et al. reported similar findings with a significant reduction in working hours with 97.9% (193/197) of neurosurgery trainees working over 60 h per week prior to the pandemic and only 34% doing the same after the pandemic began [23]. The majority of neurosurgery trainees (139/192) across Italy reported a decrease in the time they spent within the neurosurgical department, part of the strategy to reduce trainee exposure to COVID-19 [22]. A survey of 84 general surgery programme directors in the United States found that all programmes had reduced the number of trainees on daily rounds [33]. In a survey of 504 ophthalmology trainees across 32 countries, 76.4% reported more than a 50% reduction in clinical activity [34].

Trainee involvement in outpatient activity has been reduced according to many studies. In 1 study, 80.6% of general surgery trainees reported that they had not attended any outpatient activity during the pandemic [17]. In the United States, 3 out of 5 orthopaedic programmes removed trainees from all outpatient activity [31] and 86% of fellow-led head and neck clinics were cancelled during the pandemic [18]. However, remote clinical work continued to be performed by trainees in some studies [13, 26, 27]. Fifty-one percent of urology trainees (54/106) based in the United States reported that they had continued to participate in in-person clinical encounters and 65% were still participating in telehealth consultations [13]. Remote clinical work was being performed by trainees in 77% (50/65) of urology programs according to surveyed program directors in the United States [26].

### Educational/academic

Due to the decrease in clinical activity, the time for educational and academic pursuits increased. Across countries and specialities, the majority of studies reported there was a move towards online educational tools. Journal clubs, conferences and webinars were provided via virtual platforms. The proportion of those reporting a change to online education ranged from 86% [35] to 98.5% [23]. Amongst Italian general surgery trainees, the educational programme was shifted to an online system, with weekly journal club and twice weekly webinars [15].

The perception of online teaching varied. In a survey of orthopaedic trainees in Chile, 82% (82/100) viewed the new resources positively and stated that they would continue attending webinars after the pandemic [35]. Almost all (194/197) of surveyed neurosurgery trainees in the United States and Canada reported that their teaching programme had been converted to an online platform with a significantly greater proportion spending more than 4 h weekly on online learning resources than before the pandemic [23]. However, these were only received favourably by 64.6%. Paesano et al. reported that 65% (96/148) of urology trainees across Latin America and Spain felt that theoretical training has been negatively affected, this is despite 93% accessing online learning tools [27]. Similarly, Rosen et al. found that 48% (31/65) of urology programme directors in the United States reported that teaching had been negatively impacted by COVID-19 despite all surveyed programs using video-conferencing [26].

Seven studies reported that trainees now had more time to devote to educational or academic activities [13–16, 22, 23, 36]. Three studies found that the time dedicated to research had improved [13, 15, 26] and 55.7% (107/192) of neurosurgical trainees across Italy reported increased productivity of scientific papers [22].

### Wellbeing

Aside from the changes to clinical activity and working pattern, COVID-19 has had an impact on the wellbeing of surgical trainees. Over 90% of French urology trainees reported feeling more stressed than before the pandemic [37]. Factors significantly associated with stress were the presence of COVID-19 positive patients in the department (OR 2.39, 95% CI 1.30–4.39), working in a high epidemic location (OR 1.71, 95% CI 1.06–2.78) and being a more senior trainee (OR 1.76, 95% CI 1.05–2.97). More than half of surveyed ophthalmology trainees (393/716) across India also reported an increase in stress levels and 46.5% described feeling ‘unhappy’ during the lockdown period [12]. Negative impacts on mental health were reported amongst neurosurgery trainees worldwide [36] and 20% of orthopaedic trainees across 5 training programmes in the United States were using mental health resources to support their well-being during the pandemic [31]. Khusid et al. reported that performing a greater number of operations per week before the pandemic was associated with higher depression severity amongst urology trainees in the United States [28]. Thirty-three percent of 1102 general surgery trainees reported increased burnout compared to before the pandemic [17]. Only 1 study reported an improvement in mental health during the pandemic: a Pakistani study across all surgical specialities found that burnout was significantly reduced compared to pre-pandemic (*p* < 0.001) [32]. This may be due to a significant reduction in working hours.

Three studies reported that working throughout the pandemic had had an impact on life outside the hospital [13, 23, 36]. A third (62/197) of neurosurgery trainees across the United States and Canada reported that working during the pandemic had a negative impact on their interpersonal relationships [23], 54% of urology trainees felt that the pandemic had caused home-life disruption and 39% had increased worries regarding finances [13]. All of the 52 neurosurgery trainees participating in an international survey reported that their social life had been affected by the pandemic [36]. Two studies reported that trainees were concerned about the health of their loved ones or transmitting the virus to them [30, 38].

Two studies reported trainee views on obtaining employment after the end of their current rotation. Neurosurgical trainees in the United States and Canada were concerned about the change to educational experience and career prospects due to the pandemic, with 26.5% (52/197) expressing concerns over the ability to obtain employment or fellowship of their choice due to COVID-19 restrictions [23]. Amongst 51 head and neck fellows in the United States, 57% had secured a post-fellowship appointment while 10% stated that this was now in question or on hold due to COVID-19 [18].

## Discussion

COVID-19 has a negative impact across all surgical specialities and this has been felt worldwide. All studies reported a decrease in operative volume and experience for trainees with some reporting concerns over trainees’ ability to meet training requirements. There was a trend towards decreased time spent in the hospital across studies and the use of telemedicine for remote consultations. Of the studies that investigated the impact on mental health and wellbeing, all reported an adverse effect whilst working throughout the pandemic. Some positives highlighted from the studies were the move to online educational resources and additional time for self-directed study or research.

Across the 8 included surgical specialities, experience for trainees has decreased along with rising stress levels and negative mental health repercussions. With the pandemic still ongoing and the surge of a second wave, the way we deliver surgical training needs to change. Some suggestions to mitigate the loss of experience during the pandemic include a personalised approach for additional training [39], intake assessments of trainees to identify deficiencies and enable targeted interventions [39] and updating curricula to reflect the loss in opportunities [32]. Surgical simulation may also play a role in allowing trainees to gain some practical experience outside of the operating theatre [40], thereby minimising the risk of transmission of infection. It is also vital that trainees are supported from a mental health perspective during the pandemic and whilst attempting to recoup training experience.

While on a lesser scale than the current COVID-19 pandemic, the 2003 Severe Acute Respiratory Distress Syndrome (SARS) epidemic resulted in over 8000 cases across 26 countries [41]. This had an effect on medical education and health provision. Sherbino and Atzema [42] describe the impact of SARS on medical education by the loss of formal teaching sessions, delays to new clinical rotations and faculty taking on nonclinical roles so previous teaching commitments were cancelled. Toronto suffered a dramatic outbreak of SARS causing the cancellation of elective procedures and reduction in outpatient activity [43].

Surgical trainees in the United Kingdom already struggle to meet operative case requirements, with 85% coming in on days off to maximise training opportunities [44]. It is likely that the lack of operative experiences during the COVID-19 period will compound this. The lack of exposure to operative practice and resultant concerns surrounding trainees’ ability to meet minimum case requirements needs to be addressed. There is the suggestion that senior trainees may be disproportionately affected by the reduction in operative case volume and some studies report concern over future job opportunities. These findings are supported by Zheng et al. who found amongst chief residents common concerns were the inability to meet case requirements and feeling unprepared for a fellowship or future job [39].

All of the studies that investigated the effect of COVID-19 on stress, mental health and home-life disruption found a negative impact. Due to the additional stress and disruption caused by COVID-19, clinicians are at greater risk of developing burnout long-term [45]. Findings from China, reported 50.4% of health care workers reported symptoms of depression and 71.5% reported distress whilst working through the pandemic [46]. One study investigating the impact of COVID-19 on anxiety amongst medical staff in China found that female clinicians had an increased incidence of anxiety [47]. Only 1 of our studies compared degree of stress by sex, amongst French urology trainees sex was not associated with increased stress during the pandemic [37]. Surgical trainees across all specialities are already known to have high rates of burnout [48, 49], therefore it is important that trainees are given adequate support not only during but also after the pandemic.

Despite the overwhelming negative impact of COVID-19 on surgical training, some positives can be found. The rapid adaptation of educational resources to delivery through online platforms has allowed trainees to continue to develop their theoretical knowledge. Similarly, the conversion of conferences to webinar format enables a greater number of surgeons to access educational material remotely and reduces the need for study leave, costs associated with travel and accommodation [40]. Some studies reported changes to working life had allowed additional time to be devoted to research. While redeployment forces trainees to step out of their comfort zone and may cause some anxiety [50], redeployment to an appropriately supervised area can allow the acquisition of new skills and the refreshing of old [51, 52]. One of the strengths of this review is that to our knowledge this is the first systematic review on the impact of COVID-19 across all surgical specialities, focusing on trainee challenges across all the facets of surgical training. The main limitation of this study is the lack of objective data regarding the impact on operative case volume, this has prohibited a meta-analysis.

## Conclusions

The impact of COVID-19 on surgical trainees has been experienced globally and across all specialities. Negative effects are not limited to operative and clinical experience, but also the mental health and wellbeing of trainees. To quantify the true impact of COVID-19 and to make recommendations for the future provision of training, further studies using operative case volume and assessment data are required. Delivery of surgical training in the ongoing pandemic will need to move away from traditional models of learning to ensure trainees are competent and well supported.

**Table 1:** Studies that met the inclusion criteria after full-text review (*n* = 29)

Author	Country	Size	Speciality	Method	Response rate	Clinical activity impact
Abdessater	France	275	Urology	National survey	55.5%	85.5% felt the quality of their work had been affected 91.6% reported feeling stressed Risk factors for stress were past medical history (OR 2.57, 95% CI 1.31–5.98) and COVID-19 patients management (OR 1.85, 95% CI 0.98–3.59)
Alhaj	Canada, USA, Kuwait, Saudi Arabia, Serbia, Italy	52	Neurosurgery	Cross sectional survey	98.1%	98% felt training was affected 90% felt mental health was affected 100% reported their social life was affected
Amparore	Italy	351	Urology	National survey	60.8%	7.7% involved on COVID-19 wards 41.4% reduction in on call duties, 74.1% reduction in diagnostic procedures, 62.1% reduction in endoscopic surgery, 57.8% in open surgery and 44.2% for minimally invasive surgery For final year trainees, reduction from 84–44% of minimally invasive ops (*p* < 0.001) and 82 to 44% (*p* = 0.002) of major surgery 85.2% reported at least 2 h per day for smart learning purposes
An	USA	121	Orthopaedics	Survey	82%	1 out of 5 centres had redeployed trainees to support patients with COVID-19 3 out of 5 residency programmes withdrew trainees from all outpatient activity 20% using mental health resources to support wellbeing Educational activity has continued via video-conferencing
Aziz	USA	1102	General surgery	Survey	Not reported	77% reported daily rounds and floor duties were delegated to other members of staff 40.6% reported that they were not allowed in the operating room for cases considered high risk of COVID-19 transmission Significant decrease in the number of operative cases per week 80.67% attended no outpatient clinic activity during the pandemic 80.6% reported all teaching was provided by online platforms 33.1% reported more burnout than pre-COVID pandemic
Bernardi	Italy	3	General surgery	Operative log book review	N/a	Number of interventions performed significantly decreased (36.2 vs 14.0, *p* = 0.033) Mean number of surgeries as first operator decreased from 11.8 ± 7.9 to 4.5 ± 5.8 (*p* = 0.099) Non-significant trend towards decreased participation in all surgeries Education provided via webinars and other online platforms
Brar	USA	33 programme directors	Oral and maxillofacial surgery	Survey	35%	All programmes had suspended elective and non-urgent operations 40% reported using telemedicine 73% reported resources for resident wellness and stress reduction Education continued on digital platforms
Burks	USA	1 programme	Neurosurgery	Operative case review	n/a	Operative case totals were lower at all levels of training Significant decrease in cases compared to year before (*p* < 0.01)
Collins	USA	73	General and plastic surgery	Survey	73.7%	90% expressed concern about decline in operative exposure Estimated reduction in operative volume of 63.3% Residents were more concerned about the health of loved ones as opposed to their own risk of contracting COVID-19 (*p* < 0.01)
English	UK	3	General surgery	Operative log book review	N/a	Operative training continued in emergency cases Change to more open compared to laparoscopic procedures
Fero	USA	64 programme leaders, 106 trainees	Urology	Cross sectional survey	20%	20% redeployed Decreased surgical volume 83–100% 79% perceive negative impact on training Increase use of teleheath (99%), decrease size of inpatient trainee teams (90%) Transition to virtual learning
Ferrara	32 countries	504	Ophthalmology	Survey	Not reported	55.2% of trainees felt the pandemic had had a severe impact on training 76.4% reported more than a 50% reduction in clinical activity 67.7% attended web-based teaching
Figueroa	Chile	100	Orthopaedics	Survey	90.9%	86% using online educational tools 13% reported lack of practical education was a problem with online learning 30% felt all non-practical education should be performed using online tools
Givi	USA	31 trainees 37 programme directors	Otolaryngology	Survey Operative log review	Not reported	70% of program directors reported a 50–90% reduction in elective cases, but 81% stated fellows continued to participate in oncology cases 86% of fellow-led clinics cancelled Operative log comparison was similar to pre-COVID-19 times 82% of fellows have met the requisite number of operative cases for certification 97% reported weekly participation in virtual tumour board series
Guo	USA	175	Otolaryngology	Survey	83%	98.3% reported a decrease in clinical activities 68% expressed concern in the ability to receive adequate surgical training 54.7% of senior trainees felt the pandemic had had a negative impact on future job prospects
Huntley	USA	161 trainees, 13 programme directors	Oral + maxillofacial	Survey	83%	14% redeployed 96.5% reported modifications to training programme 66% not concerned with meeting graduate requirements. Greatest concern was for trainees scheduled to graduate in 2022 (60%) 88.8% felt operative experience been affected, with mean decrease in operative experience of 67% 97.7% stated elective cases stopped altogether 94.2% stated move to virtual didactics
Kapila	10 countries	86	Plastic surgery	Survey	Not reported	85% of all trainees felt their training had suffered Decreased surgical activity was reported by 86% of Belgian trainees and 73% of international trainees 46% of Belgian trainees redeployed compared to 27% of international trainees Anxiety regarding own health and that of loved ones was reported by 54% of Belgian and 69% of international trainees
Khusid	USA	332	Urology	National survey	20%	22% redeployed Factors significantly associated with concerns over future operative autonomy were ability to reach case requirements, cancellation of elective cases and PGY4 trainees Risk factors for depression were local COVID-19 severity, personal history of COVID-19 infection and concerns over operative autonomy
Mishra	India	716	Ophthalmology	Survey	Not reported	24.6% redeployed 80.7% felt training negatively impacted 75.7% thought online teaching was useful 54.8% reported higher levels of stress, 46.5% felt ‘unhappy’
Munjal	22 countries	66 residents, 30 faculty	Otolaryngology and paediatric otolaryngology	Survey	Not reported	91.7% reported all elective cases had been cancelled 86.4% felt all levels of residents were affected equally 87.5% reported that no supplementary operative education is being provided 41.7% reported that all teaching seminars are virtual
Osama	Pakistan	112	All surgical specialties	Survey	Not reported	86.6% felt hands-on experience has been adversely affected 82.1% reported clinical exposure has been affected 61% were concerned about transmitting the virus to loved ones Significant reduction in working hours per week (*p* < 0.001) Burnout was significantly reduced compared to pre pandemic (*p* < 0.001)
Paesano	Latin America Spain	148	Urology	Survey	100%	15% said urology activity completely ceased and staff redeployed 82% said activity of urology department significantly reduced 75% said surgical training has been completely affected 65% felt academic raining has been partially or completed affected
Megaloikonomos	23 European countries	327	Orthopaedics	Survey		58.6% felt surgical training was significantly impaired 58.2% were concerned about meeting annual training goals In 57.1% of institutions only essential activities were performed 20.9% redeployed
Pelargos	USA Canada	197	Neurosurgery	Survey	Not reported	35.1% providing non-specialty care to COVID-19 patients 82% inpatient and outpatient volumes reduced 91% reported work responsibility had decreased, with significant decrease in work hours (*p* < 0.001) 33.7% concerned would negatively affect residency education, with senior trainees more likely to be concerned *p* = 0.028 8.2% concerned would affect overall case numbers 26.5% concerned limit ability to get desired employment or fellowship Significant increase in number of trainees spending > 4 hours on didactics *p* < 0.0001
Rosen	USA	65 programme directors	Urology	Survey	45%	26% redeployment 60% programmes report concern trainees will not meet case minimums Reduction of trainee presence reported by 92% of programs Significant decrease in patient-contact time (4.7 to 2.1 days/week, *p* < 0.001) 48% of programmes reported education had been negatively impacted
White	USA	84 programme directors	General surgery	Survey	33.6%	44% reported attendings were operating without resident presence All programmes reduced the number of residents on rounds 90.5% reported the use of telehealth clinics 86.9% reported resident teaching moved online 8.3% reported redeployment
Zheng	USA	24	General surgery	Survey	61.5%	The biggest concern amongst chief residents was the potential delay in board examinations 93.3% of attending surgeons suggested a personalised approach for additional training
Zingaretti	Italy	115	Plastic surgery	National survey	72%	Majority feel lack of training is detrimental to professional growth The majority were using webinars to keep knowledge updated
Zoia	Italy	192	Neurosurgery	Survey	58%	72.4% reported reduced time in neurosurgical department 78.6% performed less operations, 16.1% performed no operations Production of scientific papers or research projects increased in 55.7% of cases

## Supplementary Information

Below is the link to the electronic supplementary material.

Supplementary file 1 (DOCX 18 KB)

## Data Availability

No original data were generated.
