# Supplementary material for: The impact of COVID-19 on surgical training: a systematic review
Source: Tech Coloproctol. 2021 Jan 28;25(5):505–20. doi: 10.1007/s10151-020-02404-5 (PMC7841379; doi:10.1007/s10151-020-02404-5)
Supplement: Supplementary file 1 — (DOCX 18 KB) [file 10151_2020_2404_MOESM1_ESM.docx]

Supplemental Figure 1

| 1. SARS-Cov2.mp. [mp=ti, ab, hw, tn, ot, dm, mf, dv, kw, fx, dq, nm, kf, ox, px, rx, an, ui, sy] |  |
| --- | --- |
| 2. coronavirus.mp. or Coronavirinae/ |  |
| 3. pandemic/ or covid-19.mp. |  |
| 4. covid.mp. |  |
| 5. 'surgical training'.mp. or surgical training/ |  |
| 6. 'surgeons in training'.mp. or residency education/ or surgical training/ |  |
| 7. 'surgical trainee'.mp. or surgical training/ |  |
| 8. 'surgical training programme'.mp. |  |
| 9. surgical resident.mp. or resident/ |  |
| 10. 'resident in training'.mp. |  |
| 11. postgraduate.mp. [mp=ti, ab, hw, tn, ot, dm, mf, dv, kw, fx, dq, nm, kf, ox, px, rx, ui, sy] |  |
| 12. training progression.mp. [mp=ti, ab, hw, tn, ot, dm, mf, dv, kw, fx, dq, nm, kf, ox, px, rx, ui, sy] |  |
| 13. assessment.mp. [mp=ti, ab, hw, tn, ot, dm, mf, dv, kw, fx, dq, nm, kf, ox, px, rx, ui, sy] |  |
| 14. 1 or 2 or 3 or 4 |  |
| 15. 5 or 6 or 7 or 8 or 9 or 10 |  |
| 16. training.mp. [mp=ti, ab, hw, tn, ot, dm, mf, dv, kw, fx, dq, nm, kf, ox, px, rx, ui, sy] |  |
| 17. 11 or 12 or 13 or 16 |  |
| 18. 14 and 15 and 17 |  |
